# Application of GC-MS for the detection of lipophilic compounds in diverse plant tissues

**DOI:** 10.1186/1746-4811-5-4

**Published:** 2009-04-24

**Authors:** Anna Lytovchenko, Romina Beleggia, Nicolas Schauer, Tal Isaacson, Jan E Leuendorf, Hanjo Hellmann, Jocelyn KC Rose, Alisdair R Fernie

**Affiliations:** 1 Max-Planck Institute of Molecular Plant Physiology, Am Muehlenberg 1, 14476 Potsdam-Golm, Germany; 2 CRA Cereal Research Center, S.S. 16, km 675, 71100 Foggia, Italy; 3 Department of Plant Biology, Cornell University, 331 Emerson Hall, Ithaca, New York 14853, USA; 4 Institute of Biology/Applied Genetics, Free University of Berlin, Albrecht-Thaer-Weg 6, 14195 Berlin, Germany; 5 School of Biological Sciences, Washington State University, PO Box 644236 Pullman, WA 99164-4236, USA; 6 Present address: De Ruiter Seeds, Leeuwenhoekweg 52, 2661CZ Bergschenhoek, the Netherlands

## Abstract

**Background:**

The concept of metabolite profiling has been around for decades and technical innovations are now enabling it to be carried out on a large scale with respect to the number of both metabolites measured and experiments carried out. However, studies are generally confined to polar compounds alone. Here we describe a simple method for lipophilic compounds analysis in various plant tissues.

**Results:**

We choose the same preparative and instrumental platform for lipophilic profiling as that we routinely use for polar metabolites measurements. The method was validated in terms of linearity, carryover, reproducibility and recovery rates, as well as using various plant tissues.

As a first case study we present metabolic profiling of Arabidopsis root and shoot tissue of wild type (C24) and mutant (*rsr4-1*) plants deficient on vitamin B6. We found significant alterations in lipid constituent contents, especially in the roots, which were characterised by dramatic increases in several fatty acids, thus providing further hint for the role of pyridoxine in oxidative stress and lipid peroxidation.

The second example is the lipophilic profiling of red and green tomato fruit cuticles of wild type (Alisa Craig) and the DFD (delayed fruit deterioration) mutant, which we compared and contrasted with the more focused wax analysis of these plants reported before.

**Conclusion:**

We can rapidly and reliably detect and quantify over 40 lipophilic metabolites including fatty acids, fatty alcohols, alkanes, sterols and tocopherols. The method presented here affords a simple and rapid, yet robust complement to previously validated methods of polar metabolite profiling by gas-chromatography mass-spectrometry.

## Background

In the last few years gas-chromatography mass-spectrometry has become firmly established as a key technological platform for metabolite profiling in both plant and non-plant species [1-5]. Until relatively recently only a limited number of plant research laboratories had access to gas-chromatography mass-spectrometry instrumentation, however, such machines are increasingly becoming more commonplace. The application of metabolite profiling is varied with studies ranging from the relative simplicity of diagnostics such as those used in herbicide mode-of-action studies [6], or in blood plasma analysis [7] to the complexity inherent in integrative genomics and systems biology [8-10]. In the medicinal field the majority of studies have arguably been focussed on development of metabolite profiling as a diagnostic tool. In this field particularly impressive examples have been provided by the discovery of markers for coronary heart disease and atherosclerosis [7,11]. Although early plant studies also focussed in this area (see for example [12,13]), a great deal of research is currently carried out at a more mechanistic level often encompassing other post-genomic tools. Recent examples of note in this direction are the development of combined transcript-metabolite networks for aiding functional gene annotation [14,15] and studies aimed at uncovering the genetic basis of metabolic regulation [16-20].

We have previously concentrated our own metabolite profiling activities on assessing the levels of polar primary metabolites of a wide range of species and tissues including potato (tuber and leaf), tomato (multiple tissues), strawberry (achene and receptacle), sunflower (stems), pea (embryo), medicago (roots) and cell suspension of the pennate diatom *Phaeodactylum tricornutum *[21-28]. Such measurements proved highly informative in addressing a range of questions such as defining the metabolic shifts underlying fruit development, quantifying the effects on metabolism in general of plants deficient in the expression of specific enzymes or metabolite transporters and in defining the cellular response of diatoms to iron availability.

Furthermore, our previous measurements were fully adequate for tissues, such as tomato pericarp or potato tuber, in which lipophilic components represent only a small proportion of the metabolome (when assessed on a per gram dry weight basis). However, for certain other tissues such as those of the oilseed plant Arabidopsis or specialised tissues such as the tomato fruit cuticle the data afforded by exclusive profiling of the polar metabolites are insufficient. For this reason we present here a simple yet validated and robust protocol for profiling the lipophilic components of methanol/chloroform extracts from Arabidopsis leaf and root and tomato fruit cuticles. The developed method has the additional benefit that it is reliant merely on the machinery necessary for the profiling of the polar metabolites. It should be borne in mind that with this method we measure full lipid extracts, and as such, some of the measured derivatives are actually component parts of other physiological compounds, such as membrane or storage lipids or lipid conjugates. This fact notwithstanding, this method is likely to have high utility in detecting genotypes displaying altered lipophilic profiles. We present the range of linearity and reproducibility of the method, as well as document the recovery of authentic standards added prior to extraction in order to analyse their stability throughout the processes of extraction, derivatisation, injection and analysis. In addition we present two case studies, in which we compare the lipophilic profiles of different Arabidopsis and tomato genotypes and discuss the complementarity of the method with those that we routinely use.

## Results and discussion

### Establishment of the method

The method described here was used for detection of most abundant non-polar metabolites in Arabidopsis shoot and root and tomato fruit cuticles. We evaluated forty one compounds in terms of linearity, carryover and reproducibility, and the recovery of a subset of twenty seven of the compounds covering representatives of all major compound types was additionally carried out.

We chose to develop as simple a method as possible for the detection of major non-polar compounds in different plant tissues. For this reason we adopted the exact extraction procedure routinely used in our lab for polar compound analysis (see, for example, [21,29]), thus facilitating the preparation of both polar and apolar fractions at the same time from the exact same plant material. An aliquot of the chloroform fraction, which following our usual protocol would be discarded, was then used for further analysis using the same machine settings [21]. In addition we chose to use the same retention time (RT) standards mixture of alkanes, although only the later RT standards have high utility for the analysis of the plant tissues that we have evaluated to date (since only one compound is detected between the first and third RT standard). For the sake of simplicity we also refrained from the commonly used methyl esterification procedure for fatty acids. In doing so we avoided both a time consuming and potentially dangerous chemical reaction, and in our experience freshly extracted, rapidly vacuum concentrated extracts that are derivatised shortly before analysis, display a wide spectrum of fatty acid trimethylsilyl esters (TMS), which could be reproducibly detected in various tissues.

### Validation of the method

#### Linearity

The linearity of the profiling method was assessed by analysis of standard compounds which were run in dilution series ranging from 1.25 ng to 100 ng of substance injected. The standard derivatisation protocol was used. Calibration curves were run both in ascending and descending direction, as well as completely randomised. For this purpose measurements were made in triplicate, response ratios relative to the internal standards nonadecanoic acid methyl ester were calculated, and for each metabolite the best linear correlation between response ratio and amount of the substance was determined and the linear correlation coefficient (R^2^) was calculated (Table 1; Additional files 1, 2 and 3). The values for most metabolites were greater than 0.98 for the entire range of concentrations used. Exceptions were α-tocopherol, octacosanoic acid, triacontanoic acid and α-amyrin, for which the highest concentration (100 ng) had to be omitted from the calculation, and β- and γ-tocopherol, for which two highest concentrations (50 ng and 100 ng) had to be omitted in order to achieve a good linear response. By contrast, for several metabolites, namely hentriacontane, tritriacontane, tetratriacontane and cholesterol the lowest amount (1.25 ng of substance injected) was excluded from the calculation.

**Table 1:** Method validation – linearity, reproducibility and recovery.

***Compound***	***m/z*^§^**	***RI*^#^**	***Linearity, R*^2^**	***Reproducibility, % variance***	***Recovery, %***
*n-Dodecane (C12) (RT Standard 1)*	*85*	*1200*			
*n-Pentadecane (C15) (RT Standard 2)*	*85*	*1500*			
***n*-Tetradecanoic acid (14:0) (TMS)**	242	1850	0.992	10.2	nd
*n-Nonadecane (C19) (RT Standard 3)*	*85*	*1900*			
***n*-Pentadecanoic acid (15:0) (TMS)**	299	1947	0.977	10.1	nd
***n*-Hexadecanol (1OH-16:0) (TMS)**	299	1959	0.989	10.4	105
***n*-Hexadecenoic acid (16:1) (TMS)**	311	2021	0.994	19.6	nd
***n*-Hexadecanoic acid (16:0) (TMS)**	313	2047	0.981	6.8	97
***n*-Heptadecanoic acid (17:0) (TMS)**	327	2144	0.996	9.2	nd
***n*-Octadecanol (1OH-18:0) (TMS)**	327	2154	0.991	7.1	102
*n-Docosane (C22) (Rt Standard 4)*	*85*	*2200*			
***n*-9,12-Octadecadienoic acid (18:2) (TMS)**	337	2210	0.925	17.4	nd
***n*-Octadecenoic acid (18:1) (TMS)**	339	2215	0.999	13.2	75
*n-Nonadecanoic acid methyl ester (IS)*	*269*	*2232*			
***n*-Octadecanoic acid (18:0) (TMS)**	341	2243	0.977	7.9	103
***n*-Eicosanol (TMS) (1OH-20:0)**	355	2362	0.998	5.9	91
***n*-Eicosenoic acid (20:1) (TMS)**	367	2401	0.995	14.7	nd
***n*-Eicosanoic acid (20:0) (TMS)**	369	2454	0.989	19.1	84
***n*-Docosanoic acid (22:0) (TMS)**	397	2649	0.996	15.6	nd
***n*-Heptacosane (C27)**	380	2708	0.976	7.7	97
*n-Octacosane (C28) (Rt Standard 5)*	*85*	*2800*			
***n*-Tetracosanoic acid (24:0) (TMS)**	425	2838	0.994	14.7	77
**δ-tocopherol (TMS)**	474	2900	0.998	17.7	75
***n*-Nonacosane (C29)**	408	2902	0.983	12.2	96
***n*-Hexacosanol (TMS) (1OH-26:0)**	439	2945	0.985	5.1	nd
**β-tocopherol (TMS)**	488	2987	0.999^a^	19.8	79
**γ-tocopherol (TMS)**	488	2999	0.926^a^	14.1	83
***n*-Triacontane (C30)**	422	3006	0.981	15.3	90
***n*-Hexacosanoic acid (26:0) (TMS)**	468	3037	0.925	9.7	91
***n*-Heptacosanol (TMS) (1OH-27:0)**	453	3043	0.986	10.9	nd
***n*-Hentriacontane (C31)**	436	3105	0.964^b^	10.1	97
***n*-Octacosanol (TMS) (1OH-28:0)**	467	3139	0.985	11.9	88
**α-tocopherol (TMS)**	502	3143	0.998^c^	7.9	82
**Cholesterol (TMS)**	458	3155	0.983^b^	13.3	nd
*n-Dotriacontane (C32) (Rt Standard 6)*	*85*	*3200*			
***n*-Nonacosanol (TMS) (1OH-29:0)**	481	3236	0.917	6.7	nd
**Campesterol (TMS)**	382	3263	0.989	6.9	102
***n*-Octacosanoic acid (28:0) (TMS)**	481	3269	0.977^c^	10.5	83
**Stigmasterol (TMS)**	484	3286	0.989	5.7	92
***n*-Tritriacontane (C33)**	464	3304	0.956^b^	9.1	91
***n*-Triacontanol (TMS) (1OH-30:0)**	495	3337	0.989	8.8	nd
**β-Sitosterol (TMS)**	396	3344	0.997	8.1	90
**δ-amyrin (TMS)**	189	3375	0.993	19.1	nd
**β-amyrin (TMS)**	498	3385	0.989	17.9	89
***n*-Triacontanoic acid (30:0) (TMS)**	509	3412	0.933^c^	19.2	76
**α-amyrin (TMS)**	498	3429	0.992^c^	15.5	80
***n*-Tetratriacontane (C34)**	478	3463	0.981^b^	18.2	nd
***n*-Dotriacontanol (TMS) (1OH-32:0)**	523	3533	0.967	9.9	98
*n-Hexatriacontane (C36) (RT Standard 7)*	*85*	*3600*			

nd – not determined; IS – internal standard

^§ ^specific mass ion used for quantification. Full spectrum information could be found at GMD database [55] and in Additional files 1, 2 and 3 (for α-amyrin, δ-amyrin and *n*-eicosenoic acid)

^# ^relative retention index based on interpolation of retention times between alkane retention standards

^a ^– range 1.25–25 ng injected

^b ^– range 6.25–100 ng injected

^c ^– range 1.25–50 ng injected

#### Reproducibility

Both sample preparation procedure and instrumental analysis contribute to the variability of the method. The overall reproducibility of the method was tested by analysis of ten replicate extracts of 100 mg of the same sample of Arabidopsis leaf (ecotype Col-0) or 50 mg of red tomato cuticles (cultivar Alisa Craig) for assessment of behaviour of tocopherols and amyrins, where they were highly abundant. Samples were all run on the same day. Variability values (standard deviations) presented on a percent basis are listed in Table 1. For most metabolites values were between 5 and 15%, but for quarter of evaluated compounds they were slightly higher, between15 and 20%. These levels of variability are broadly in agreement with previously reported values for metabolite profiling protocols for a range of compound classes and instrumentation (see, for example, [21,30,31]).

#### Recovery

For the assessment of the extraction procedure and the chemical stability of the metabolites/derivatives recovery experiments were conducted. For this purpose double the amounts, present in the extract, of several authentic standard compounds were added at the beginning of extraction procedure to the tissue and recovery rates were calculated by running alongside unspiked extracts from the same biological sample. Given the prominence of Arabidopsis in genomics research we chose to perform these experiments in leaf tissue of this species (with exception of tocopherols and amyrins, for which red tomato cuticles were used). For all tested metabolites recovery values were in the range of 75–105% which is in close agreement with previous reports for polar metabolite determination via GC-MS (see, for example, [21])

To assess the applicability of the method for various plant tissues, recombination experiments were performed, in which aliquot of Arabidopsis leaf tissue was mixed with an aliquot of all other tissues and extracted in parallel with both tissues separately. Following analysis of these samples chromatograms were checked manually for possible peak shifts, for instances of peak co-elution and the appearance of new peaks, and the peak finding method was corrected accordingly as we have routinely done on optimisation of the polar method for novel tissues (see [32] for a detailed description).

#### Carryover

As a further validation we evaluated carryover rate which is noted as problematic for lipophilic compounds. For this purpose we assessed the peak area of each analyte in chromatograms of plant extract followed by blank injections (washes). These studies revealed that carryover was generally quite low with 31 of 41 compounds displaying carryover rate lower than 15%, nine between 15 and 20% and only one compound (alkane *n*-tritriacontane) showed carryover rate of 21.8% [see Additional file 4].

As an initial case study we chose to analyse both leaf and root samples of an Arabidopsis mutant deficient in pyridoxine (Table 2). We have previously characterised this *rsr4-1 *mutant, which is deficient in vitamin B6 (pyridoxine) biosynthesis, as displaying broad alterations in metabolism with the levels of the polar metabolites of the amino acid and organic acid compound classes being most affected as well as the levels of raffinose and shikimate [33]. Given that the phosphorylated form of vitamin B6 is a co-factor not only in reactions associated with amino acid but also those associated with lipid degradation [34,35] and is furthermore believed to act as a protective agent against reactive oxygen species, such as singlet oxygen [36,37], we thought it would be interesting to look at the lipophilic profiles of this mutant. With this aim in mind we harvested material from roots and leaves of six individuals from both the wild type and *rsr4-1 *mutant. Although not reported in the previous manuscript, the roots of this mutant also displayed marked changes in amino acids (Lytovchenko A, Leuendorf JE, Hellmann H, Fernie AR, unpublished data). Intriguingly, the roots displayed massive alterations in their lipophilic profiles with 18 or the 22 metabolites measured exhibiting significantly increased levels and with some of these, such as eicosanoic acid and α-tocopherol displaying in excess of 5-fold increases. In the leaves of the mutant, the lipophilic components were, however, much more similar to those of the wild type with only three compounds (hexadecanol, hexacosanol and triacontanol) displaying altered levels in the mutant. In contrast to the situation seen in the leaves, in each instance the metabolite levels were decreased. That lipophilic metabolites are altered in the *rsr4-1 *mutant is not without precedence, since there is a long history of research of the role of pyridoxine on fatty acid metabolism. As early as 1952 it was suggested to be important for the linoleate to arachidonate conversion and it has also previously conversely been speculated that it could be involved in the catabolism of arachidonate [38]. However, there is to date no direct experimental evidence that confirms either of these postulates. Intriguingly, rats fed with a pyridoxine deficient diet (rats cannot synthesize the vitamin *de novo *but have an effective salvage pathway that converts pyridoxine to PLP), also have a tendency of increased unsaturated fatty acids [39]. In contrast, such rats have also been characterized to be deficient in galactolipids [40]. Despite these interesting observations, the only direct link between PLP and fatty acid biosynthesis appears to be its role in sphingolipid synthesis [41], although it is possible to speculate that some of the changes we observed are the consequence of a reduced rate of biotin biosynthesis, which is PLP- dependent [42], since the Acetyl CoA carboxylase is a biotin dependent enzyme [43]. However, as yet we have no evidence in support of this theory. Interestingly, the response of the lipophilic profiles of root and leaf of the *rsr*4 mutant were greatly different. The exact reasons underlying these differences are, however, not apparent from the present study. A recent study by Chen and Xiong [37] revealed, that the null mutant for the *pdx1.3 *gene (which is allelic to *rsr4*), was hypersensitive to UV treatment and other stresses, however, the authors did not measure the fatty acid content. It is conceivable, that the changes in the metabolic profile of the root were indicative of oxidative stress, however, we cannot exclude the fact that they may also be caused by developmental abnormalities in the roots. Whilst not allowing a precise mechanistic insight into this pathway, these data do provide highly interesting leads for future research.

**Table 2:** Lipophilic metabolite contents in roots and shoots of agar-grown seedlings of *rsr4-1 *Arabidopsis mutant.

	***WT***	***rsr4-1***
**Roots**		
14:0	1.00 ± 0.05	**1.80 ± 0.12**
15:0	1.00 ± 0.05	**1.79 ± 0.09**
16:0	1.00 ± 0.04	**1.84 ± 0.15**
17:0	1.00 ± 0.04	**1.87 ± 0.12**
18:0	1.00 ± 0.05	**1.87 ± 0.15**
18:1	1.00 ± 0.07	**2.19 ± 0.19**
20:0	1.00 ± 0.06	**2.91 ± 0.13**
20:1	1.00 ± 0.10	**8.08 ± 0.35**
22:0	1.00 ± 0.06	**2.05 ± 0.12**
24:0	1.00 ± 0.12	**3.07 ± 0.19**
26:0	1.00 ± 0.11	**3.91 ± 0.23**
1OH-16:0	1.00 ± 0.06	**1.65 ± 0.14**
1OH-18:0	1.00 ± 0.06	**1.49 ± 0.10**
1OH-20:0	1.00 ± 0.15	1.58 ± 0.19
1OH-28:0	1.00 ± 0.21	**3.40 ± 0.26**
1OH-30:0	1.00 ± 0.15	**3.72 ± 0.27**
C33	1.00 ± 0.12	**2.02 ± 0.21**
C34	1.00 ± 0.05	**1.68 ± 0.14**
α-tocopherol	1.00 ± 0.25	**5.90 ± 0.27**
β-sitosterol	1.00 ± 0.12	1.11 ± 0.13
campesterol	1.00 ± 0.15	0.93 ± 0.16
stigmasterol	1.00 ± 0.16	0.89 ± 0.16
**Shoots**		
14:0	1.00 ± 0.07	0.85 ± 0.09
15:0	1.00 ± 0.07	0.87 ± 0.06
16:0	1.00 ± 0.07	0.88 ± 0.06
17:0	1.00 ± 0.08	0.85 ± 0.07
18:0	1.00 ± 0.06	0.89 ± 0.05
18:1	1.00 ± 0.06	0.93 ± 0.10
18:2	1.00 ± 0.14	1.02 ± 0.18
20:0	1.00 ± 0.06	0.94 ± 0.07
20:1	1.00 ± 0.06	2.09 ± 0.21
22:0	1.00 ± 0.10	0.80 ± 0.09
24:0	1.00 ± 0.02	0.89 ± 0.05
26:0	1.00 ± 0.04	0.94 ± 0.04
28:0	1.00 ± 0.06	0.98 ± 0.06
30:0	1.00 ± 0.07	0.96 ± 0.06
1OH-16:0	1.00 ± 0.07	**0.82 ± 0.05**
1OH-18:0	1.00 ± 0.04	0.91 ± 0.05
1OH-20:0	1.00 ± 0.03	0.92 ± 0.05
1OH-26:0	1.00 ± 0.10	**0.67 ± 0.05**
1OH-30:0	1.00 ± 0.06	**0.72 ± 0.08**
1OH-32:0	1.00 ± 0.07	0.93 ± 0.08
C27	1.00 ± 0.06	0.99 ± 0.05
C34	1.00 ± 0.14	0.93 ± 0.18
α-tocopherol	1.00 ± 0.04	0.99 ± 0.07
β-tocopherol	1.00 ± 0.06	0.94 ± 0.05
δ-tocopherol	1.00 ± 0.06	0.86 ± 0.08
β-sitosterol	1.00 ± 0.04	1.00 ± 0.06
campesterol	1.00 ± 0.04	1.02 ± 0.06
cholesterol	1.00 ± 0.03	0.82 ± 0.19
stigmasterol	1.00 ± 0.07	0.95 ± 0.05

Values are expressed relative to wild type and presented as mean ± SE of determinations from six independent samples. Those determined by the t-test to be significantly different from wild type are set in bold type.

As a second example we next evaluated the lipophilic content in enzymatically isolated cuticle tissue of both developing (green) and mature (red) fruits of Alisa Craig and the DFD (delayed fruit deterioration) mutant/accession of tomato. The levels of epicuticular waxes of these genotypes have previously been reported for these tissues of both genotypes [44]. Since the documented data was obtained using a different analytical method, we thought it would be highly interesting to evaluate this tissue using the method we developed here and to compare the results. For this purpose we evaluated cuticle preparation from fruits of five independent plants from both genotypes at both developmental stages. The changes in the absolute amounts of the various classes were fairly consistent between the studies, albeit far more of the changes observed were statistically significant in the current study. A further difference was that in the initial study there were no major differences in the individual wax constituent percentages suggesting little difference in their absolute amounts; however, in the data presented here there are significant changes in the relative levels of some but not all of the lipophilic compounds in each chemical class. Some of this discrepancy can clearly be explained by the fact that there is a general tendency for increases across the compound class, such as those observed for sterols and terpenoids, as well as for alkanes. However, for the fatty acids and fatty acid alcohols the differences in magnitude between the changes of independent members of each compound class are too great to account for the apparent discrepancy, suggesting that some of those may result from the differences in sample preparations.

In green fruit we determined the levels of 40 metabolites and found that 22 of these were significantly elevated in cuticles isolated from green fruit of the DFD mutant (Table 3).

**Table 3:** Lipophilic metabolite contents in green cuticles of DFD mutant and Alisa Craig wild type tomato.

	***WT green***	***DFD green***
**Fatty acids**		
14:0	1.00 ± 0.29	**5.82 ± 0.18**
16:0	1.00 ± 0.46	**6.68 ± 0.16**
17:0	1.00 ± 0.44	**5.15 ± 0.16**
18:0	1.00 ± 0.47	**5.04 ± 0.17**
18:1	1.00 ± 0.24	1.67 ± 0.15
18:2	1.00 ± 0.37	1.70 ± 0.18
20:0	1.00 ± 0.24	0.59 ± 0.05
22:0	1.00 ± 0.19	1.65 ± 0.13
24:0	1.00 ± 0.23	**2.48 ± 0.15**
26:0	1.00 ± 0.19	**5.68 ± 0.12**
**Fatty acid alcohols**		
1OH-16:0	1.00 ± 0.31	1.13 ± 0.24
1OH-18:0	1.00 ± 0.18	1.42 ± 0.10
1OH-20:0	1.00 ± 0.15	**3.43 ± 0.11**
1OH-26:0	1.00 ± 0.16	**18.14 ± 0.13**
1OH-27:0	1.00 ± 0.18	**17.64 ± 0.09**
1OH-28:0	1.00 ± 0.27	**13.44 ± 0.16**
1OH-29:0	1.00 ± 0.37	**8.91 ± 0.09**
1OH-30:0	1.00 ± 0.39	**6.64 ± 0.24**
1OH-32:0	1.00 ± 0.40	**2.71 ± 0.12**
**Alkanes**		
C22^a^	1.00 ± 0.39	3.61 ± 0.30
C27	1.00 ± 0.27	**7.95 ± 0.17**
C28^a^	1.00 ± 0.12	**3.93 ± 0.16**
C29	1.00 ± 0.17	**6.04 ± 0.12**
C30	1.00 ± 0.23	1.75 ± 0.20
C31	1.00 ± 0.39	**3.03 ± 0.20**
C32^a^	1.00 ± 0.27	3.97 ± 0.71
C33	1.00 ± 0.35	1.47 ± 0.21
**Sterols and terpenoids**		
α-amyrin	1.00 ± 0.25	1.79 ± 0.15
β-amyrin	1.00 ± 0.33	1.20 ± 0.14
δ**-**amyrin	1.00 ± 0.19	**2.44 ± 0.15**
β-sitosterol	1.00 ± 0.10	**1.84 ± 0.14**
campesterol	1.00 ± 0.12	**1.63 ± 0.12**
cholesterol	1.00 ± 0.10	1.27 ± 0.18
multiflorenol^b^	1.00 ± 0.25	1.31 ± 0.16
stigmasterol	1.00 ± 0.12	1.15 ± 0.14
taraxasterol^b^	1.00 ± 0.24	1.51 ± 0.14
α-tocopherol	1.00 ± 0.22	**4.04 ± 0.28**
β-tocopherol	1.00 ± 0.19	2.24 ± 0.20
γ-tocopherol	1.00 ± 0.19	**2.52 ± 0.17**
δ-tocopherol	1.00 ± 0.23	1.12 ± 0.09

Values are expressed relative to wild type and presented as mean ± SE of determinations from six independent samples. Those determined by the t-test to be significantly different from wild type are set in bold type.

^a ^were determined in a parallel separate run without retention time standards mixture

^b ^were identified according to [52]

Some of these changes were dramatic with over five fold increases in tetradecanoic, hexadecanoic, heptadecanoic, octadecanoic and hexacosanoic acids; as well as in nonacosanol, triacontanol, heptacosane and nonacosane; and over 13-fold increases in hexacosanol, heptacosanol and octacosanol. A similar picture was also obtained for the red fruit (Table 4), but in this case 37 of the 40 metabolites were significantly elevated, two unchanged and as previously commented on [44], one – naringenin chalcone decreased dramatically to levels below the detection limit. In this tissue it is quite possible that evaluating changes in % composition masked some of the changes observed, but it is also worth noting that the standard errors associated with the previous method were also very high, which could additionally obscure changes. That said it must be stated, that several of the changes recorded here were dramatic with those in tetracosanoic acid, dotriacontanol, nonacosanol and α-tocopherol being above ten-fold, and in octacosanol, nonacosanol and triacontanol above 15-fold. Of particular note is the massive change in α-tocopherol content which could be of high potential interest for researchers interested in breeding high nutrient crops. Given the ease of compatibility of this method with previously described polar profiling methods, it may well prove highly useful in future biochemical characterisations of large mutant populations which are, by and large, reliant on simple and rapid screening methodologies.

**Table 4:** Lipophilic metabolite contents in red cuticles of DFD mutant and Alisa Craig wild type tomato.

	***WT red***	***DFD red***
**Fatty acids**		
14:0	1.00 ± 0.18	**4.34 ± 0.12**
15:0	1.00 ± 0.16	**5.97 ± 0.14**
16:0	1.00 ± 0.24	**4.07 ± 0.32**
17:0	1.00 ± 0.10	**1.76 ± 0.04**
18:0	1.00 ± 0.21	**2.64 ± 0.31**
18:1	1.00 ± 0.37	2.27 ± 0.20
18:2	1.00 ± 0.09	**5.96 ± 0.07**
20:0	1.00 ± 0.21	1.34 ± 0.16
22:0	1.00 ± 0.10	**8.95 ± 0.19**
24:0	1.00 ± 0.12	**13.10 ± 0.15**
**Fatty acid alcohols**		
1OH-16:0	1.00 ± 0.07	**3.57 ± 0.22**
1OH-18:0	1.00 ± 0.09	**2.30 ± 0.15**
1OH-20:0	1.00 ± 0.05	**4.38 ± 0.29**
1OH-26:0	1.00 ± 0.11	**8.95 ± 0.21**
1OH-28:0	1.00 ± 0.08	**16.23 ± 0.13**
1OH-29:0	1.00 ± 0.10	**15.52 ± 0.12**
1OH-30:0	1.00 ± 0.10	**22.53 ± 0.13**
1OH-32:0	1.00 ± 0.14	**10.53 ± 0.18**
**Alkanes**		
C22^a^	1.00 ± 0.17	**3.96 ± 0.10**
C27	1.00 ± 0.08	**7.09 ± 0.24**
C28^a^	1.00 ± 0.12	**4.69 ± 0.18**
C29	1.00 ± 0.08	**12.00 ± 0.11**
C30	1.00 ± 0.11	**6.56 ± 0.15**
C31	1.00 ± 0.12	**7.49 ± 0.16**
C32^a^	1.00 ± 0.14	**4.01 ± 0.05**
C33	1.00 ± 0.15	**2.76 ± 0.18**
**Sterols and terpenoids**		
α-amyrin	1.00 ± 0.09	**5.42 ± 0.13**
β-amyrin	1.00 ± 0.08	**4.02 ± 0.12**
δ**-**amyrin	1.00 ± 0.07	**5.53 ± 0.13**
β-sitosterol	1.00 ± 0.12	**8.52 ± 0.09**
campesterol	1.00 ± 0.10	**8.79 ± 0.08**
cholesterol	1.00 ± 0.09	**6.17 ± 0.17**
multiflorenol^b^	1.00 ± 0.06	**5.13 ± 0.11**
naringenin-chalcone^b^	1.00 ± 0.56	**nd**
stigmasterol	1.00 ± 0.10	**4.20 ± 0.11**
taraxasterol^b^	1.00 ± 0.07	**6.18 ± 0.12**
α-tocopherol	1.00 ± 0.12	**12.25 ± 0.29**
β-tocopherol	1.00 ± 0.12	**5.62 ± 0.08**
γ-tocopherol	1.00 ± 0.15	**6.10 ± 0.19**
δ-tocopherol	1.00 ± 0.25	**4.97 ± 0.24**

Values are expressed relative to wild type and presented as mean ± SE of determinations from six independent samples. Those determined by the t-test to be significantly different from wild type are set in bold type.

^a ^were determined in a parallel separate run without retention time standards mixture

^b ^were identified according to [52]

## Conclusion

The method presented here affords a simple and rapid yet robust complement to previously validated methods of polar metabolite profiling by gas-chromatography mass-spectrometry. Given the high turnover rates of metabolites, it is imperative that methods are developed in a way that clearly demonstrates and documents their analytic precision [45]. The method presented here is generally similar to that published for potato metabolomics during the course of our study [30]. Whilst the potato study covers a slightly different range of metabolites than ours, the general analytic performance of the techniques is by and large comparable. Taking all our data into account, we conclude that we can rapidly and reliably detect and quantify over 40 lipophilic metabolites including fatty acids, fatty alcohols, alkanes and lipophilic vitamins. Whilst there has been much recent focus on standards initiatives in metabolomics [46,47], relatively few studies tackle the issues of metabolite stability through the extraction, derivatisation and analytical processes [48]. Here we have provided recoveries across the specific compound classes measured as well as confirming peak identification in the different samples via mixing or "recombination" experiments. These experiments revealed that the method was appropriate for the four tissues described here; however, we caution that such experiments should be empirically repeated for each new tissue analysed. In applying the method to two previously studied genotypes we were able to confirm its validity, as well as to extend the biochemical characterisation of these genotypes. The study on tomato cuticles has been largely consistent with the previous evaluations of this tissue using other analytical techniques [44,49]. That on the *rsr4-1 *mutant of Arabidopsis, however, provided novel data revealing comprehensive changes in the lipophilic profiles consistent with the suggested role of PLP both as a co-factor in lipid degradation and in the cellular response to oxidative stress. As such these case studies highlight the general utility of this method in expanding our coverage of the small molecular weight metabolome of plants.

## Methods

### Plant material

#### Arabidopsis

Arabidopsis 7-day-old seedlings of wild type (C24) and mutant (*rsr4-1*) plants [33] were grown under sterile conditions on vertically positioned agar plates containing basic Ats medium [50] without additional supplementation of sucrose. Plants were grown in a 16:8 day: night rhythm at 20 degrees Celsius using Phillips TLD 36W 1/830 light bulbs (150 μmol*m^-2 ^*s^-1 ^PAR at height of the growing seedlings). After 7d plants were carefully removed from plates 8 h after light onset. Shoot and root tissues were separated from each other with a scalpel and immediately weighted on a Sartorius LE224S fine scale (Sartorius AG Göttingen). For metabolic analysis, between 100–150 mg and 50–100 mg of tissue were harvested for C24 and *rsr4-1*, respectively. Samples were immediately shock frozen in liquid nitrogen. Results are based on six independent samples.

#### Tomato

Tomato fruits were grown and harvested at the mature green and red ripe stages as described in [44]. Cuticles were enzymatically isolated [51] from tomato fruit exocarp discs by incubating at 32°C in mixture of cellulose (40 U/ml) and pectinase (10 U/ml) in sodium citrate buffer (50 mM, pH 4.0), 1 mM NaN_3 _for 7 to 10 days. Cuticles were washed in distilled water and incubated again in the enzymatic buffer until clean. Cuticles were then washed in distilled water and dried at room temperature.

### Chemicals

All chemicals and pure standard compounds were purchased from Sigma-Aldrich (Deisenhofen, Germany), with exception of trans-9-hexadecenoic acid, campesterol, β-sitosterol and stigmasterol, which were from Biotrend (Cologne, Germany) and N-Methyl-N-(trimethylsilyl)trifluoroacetamide (MSTFA) from Macherey-Nagel (Dueren, Germany). Solvents were of liquid chromatography grade and were supplied by Merck (Darmstadt, Germany).

### Extraction and derivatisation of the samples

Extraction and derivatisation of the plant samples were done essentially as in [29], with exception that different volumes of solvents were used according to the fresh weight of the samples, and different aliquots of non-polar (chloroform) phase were taken for derivatisation. Briefly, Arabidopsis shoots (about 100 mg fresh weight) and roots (about 70 mg fresh weight) were homogenized and extracted in 1400 μl of 100% methanol with addition of 60 μl of nonadecanoic acid methyl ester (internal quantitative standard, 0.2 mg ml^-1 ^stock in chloroform) at 70°C for 15 min, then centrifuged, supernatant transferred to glass vial and double distilled water (1000 μl) and chloroform (1000 μl) were added, tubes were vigorously shaked and centrifuged. Aliquots of 400 μl chloroform phase for shoots and roots were dried in speed-vac and derivatised afterwards. Tomato fruit cuticles (20–50 mg dry weight) were homogenized with pestle and mortar and extracted in 3000 μl of methanol, 1000 μl of water and 2000 μl of chloroform with 60 μl of nonadecanoic acid methyl ester. Aliquots of 1200 μl of non-polar phase were dried and used for further analysis.

For derivatisation 70 μl of MSTFA reagent for Arabidopsis shoots, 35 μl for roots and 50 μl for tomato cuticles together with 12 μl of retention time standards mixture (alkanes dissolved in pyridine (see Table 1, Standards 1 and 2 - 2 μl per ml stock, Standards 3–7 2 mg per ml stock, then mixed in equal amounts) were added and incubated at 37°C for 30 min. As alternatives, fatty acid methyl esters (FAMEs) or isotope labelled retention time markers could also be used [see, for example, [12,29]]. All reagents and retention time standards were used routinely for both polar and non-polar phase analysis.

### Linearity experiment

Linearity of the response of different amounts of metabolites was checked by injection of dilution series of standard compounds dissolved in chloroform (usually 1 mg ml^-1 ^stock). The amounts of all metabolites tested were 1.25, 6.25, 12.5, 25, 50 and 100 ng of each injected substance.

### Reproducibility experiment

Ten aliquots of approximately 100 mg of Arabidopsis (Col-0) leaf tissue (the same pooled frozen material) were extracted and run at the same day. For the evaluation of amyrins and tocopherols tomato (cv. Alisa Craig) red cuticle tissue aliquots (50 mg) was used.

### Recovery experiment

For estimation of efficiency of extraction procedure, the recovery rates of various metabolites were determined. To this end concentrations of endogenous compounds were determined in 100 mg of Arabidopsis leaf tissue (for recovery of amyrins and tocopherols tomato (cv. Alisa Craig) red cuticle tissue was used, 50 mg) and then double amounts of standard compounds were added at the start of extraction procedure.

### Carryover experiment

Carryover rate was determined for each individual metabolite measured. For this purpose we assessed the peak areas of each analyte in the chromatograms of plant extract followed by blank injections of MSTFA. For statistical analysis at least 3 replicates were used.

### GC-MS analysis

Sample analysis was performed essentially as described in [21] with the exception of the column used (suitable both for polar and non-polar fraction analysis). Briefly, sample volumes of 1 μl were injected with a split ratio of 25:1 using a hot-needle technique. The GC-MS system consisted of an AS 2000 autosampler, a GC 8000 gas chromatograph and a Voyager quadrupole mass spectrometer (ThermoQuest, Manchester, UK). Gas chromatography was performed on a 30 m capillary column Rtx-5Sil MS of 0.25 mm inner diameter with integrated guard column and a 0.25 μm film (Restek GmbH, Bad Homburg, Germany). Injection temperature was 230°C, the interface set to 250°C and the ion source adjusted to 200°C. The carrier gas used was helium set at a constant flow rate of 1 mlmin^-1^. The temperature program was 5 min isothermal heating at 70°C, followed by a 5°C min^-1 ^oven temperature ramp to 310°C and a final 1 min heating at 310°C. The system was then temperature equilibrated for 6 min at 70°C prior to injection of the next sample. Mass spectra were recorded at two scans per sec with an *m*/*z *50–600 scanning range. The chromatograms and mass spectra were evaluated using the Masslab software (ThermoQuest, Manchester, UK).

### Data analysis

Specific ions characteristic of each metabolite were selected and time windows were defined relative to an adjacent retention time standards for compound detection in processing methods created using Masslab. Processed data were checked manually and corrected when necessary before subjecting to further data analysis. Compounds were identified by using standard substances analysis, comparison with MS libraries and literature data (for example, [52-55]) and by extrapolation from data for known compounds (for example, for alkanes which have very characteristic and similar spectra). Processed data were subjected to statistical analysis (the term significant is used only when p < 0.05 according to the *t*-test embedded in Excel).

## Competing interests

The authors declare that they have no competing interests.

## Authors' contributions

AL and RB carried out the experimental work with support from NS, TI and JEL. ARF, HH and JKCR conceived the study and devised the experimental design. AL and ARF wrote the manuscript. All authors read and approved the final manuscript.

## Supplementary Material

Additional File 1 **α-amyrin spectrum**. The data provided represent the list of m/z of α-amyrin with absolute and relative intensities. Click here for file

Additional File 2 **δ-amyrin spectrum**. The data provided represent the list of m/z of δ-amyrin with absolute and relative intensities. Click here for file

Additional File 3 ***n*-eicosenoic acid spectrum**. The data provided represent the list of m/z of *n*-eicosenoic acid with absolute and relative intensities. Click here for file

Additional File 4 **Evaluation of carryover**. The data provided represent estimation of carryover for each lipophilic compound from this study in percents. Click here for file
