# Supplementary material for: Application of GC-MS for the detection of lipophilic compounds in diverse plant tissues
Source: Plant Methods. 2009 Apr 24;5:4. doi: 10.1186/1746-4811-5-4 (PMC2680844; doi:10.1186/1746-4811-5-4)
Supplement: Additional File 4 — Evaluation of carryover. The data provided represent estimation of carryover for each lipophilic compound from this study in percents. [file 1746-4811-5-4-S4.doc]

**Additional file 4 –** **Evaluation of carryover**

Percentage of carryover was determined by repetitive injection of plant extracts followed by blank controls and estimation of corresponding peak areas in sample and blank chromatograms. Data are presented as average of at least three replicates for each compound

| ***Compound*** | ***carryover, %*** |
| --- | --- |
| *n*-Tetradecanoic acid (14:0) (TMS) | 5.1 |
| *n*-Pentadecanoic acid (15:0) (TMS) | 10.5 |
| *n*-Hexadecanol (1OH-16:0) (TMS) | 4.3 |
| *n*-Hexadecenoic acid (16:1) (TMS) | 9.0 |
| *n*-Hexadecanoic acid (16:0) (TMS) | 15.7 |
| *n*-Heptadecanoic acid (17:0) (TMS) | 7.0 |
| *n*-Octadecanol (1OH-18:0) (TMS) | 3.5 |
| *n*-9,12-Octadecadienoic acid (18:2) (TMS) | 18.3 |
| *n*-Octadecenoic acid (18:1) (TMS) | 10.1 |
| *n*-Octadecanoic acid (18:0) (TMS) | 3.0 |
| *n*-Eicosanol (TMS) (1OH-20:0) | 0.5 |
| *n*-Eicosenoic acid (20:1) (TMS) | 6.3 |
| *n*-Eicosanoic acid (20:0) (TMS) | 2.7 |
| *n*-Docosanoic acid (22:0) (TMS) | 7.7 |
| *n*-Heptacosane (C27) | 12.7 |
| *n*-Tetracosanoic acid (24:0) (TMS) | 14.1 |
| δ-tocopherol (TMS) | 3.9 |
| *n*-Nonacosane (C29) | 14.5 |
| *n*-Hexacosanol (TMS) (1OH-26:0) | 6.4 |
| β-tocopherol (TMS) | 1.0 |
| γ-tocopherol (TMS) | 3.7 |
| *n*-Triacontane (C30) | 15.5 |
| *n*-Hexacosanoic acid (26:0) (TMS) | 12.6 |
| *n*-Heptacosanol (TMS) (1OH-27:0) | 16.3 |
| *n*-Hentriacontane (C31) | 18.1 |
| *n*-Octacosanol (TMS) (1OH-28:0) | 12.6 |
| α-tocopherol (TMS) | 0.8 |
| Cholesterol (TMS) | 2.3 |
| *n*-Nonacosanol (TMS) (1OH-29:0) | 9.4 |
| Campesterol (TMS) | 2.4 |
| *n*-Octacosanoic acid (28:0) (TMS) | 11.1 |
| Stigmasterol (TMS) | 2.3 |
| *n*-Tritriacontane (C33) | 21.8 |
| *n*-Triacontanol (TMS) (1OH-30:0) | 15.7 |
| β-Sitosterol (TMS) | 3.1 |
| δ-amyrin (TMS) | 0.9 |
| β-amyrin (TMS) | 1.3 |
| *n*-Triacontanoic acid (30:0) (TMS) | 8.1 |
| α-amyrin (TMS) | 1.4 |
| *n*-Tetratriacontane (C34) | 17.7 |
| *n*-Dotriacontanol (TMS) (1OH-32:0) | 19.1 |
